# Analysis of Implementation Strategies for Nationwide HPV Vaccination Programs Across European Union Countries

**DOI:** 10.3390/vaccines12121325

**Published:** 2024-11-26

**Authors:** Wojciech Miazga, Tomasz Tatara, Mariusz Gujski, Janusz Ostrowski, Jarosław Pinkas, Urszula Religioni

**Affiliations:** 1 School of Public Health, Centre of Postgraduate Medical Education of Warsaw, Kleczewska 61/63, 01-826 Warsaw, Poland; 2 Department of Public Health, Faculty of Health Sciences, Medical University of Warsaw, 02-091 Warsaw, Poland

**Keywords:** national immunization program, HPV, human papillomavirus, vaccine, population

## Abstract

Background/Objectives: Human papillomavirus (HPV) vaccination programs play a critical role in the primary prevention of HPV-related diseases, including cervical cancer. However, the principles governing the implementation of these programs vary across European Union (EU) countries. The objective of this study was to analyze and compare the strategies for implementing HPV vaccination programs across the EU, with a focus on access, vaccine selection, and procurement processes. Methods: This study utilized a comprehensive review of official websites from government bodies, public health organizations, and dedicated vaccination platforms in each of the 27 EU member states. Additionally, a search of Tenders Electronic Daily (TED) was conducted to examine the criteria used in tendering processes for vaccine suppliers involved in national HPV vaccination programs. Results: All 27 EU countries provide public funding for HPV vaccination, with 26 countries offering free vaccination for both girls and boys. In 22 of these countries, the nine-valent HPV vaccine is the only option available for free under national programs. Estonia and Ireland are exceptions, where a single dose of Gardasil 9 is administered. Most countries adhere to the approved vaccination schedules, though slight variations exist. The predominant criterion for selecting vaccine suppliers in national tenders is the lowest price offered. Conclusions: HPV vaccination programs across the EU are uniformly funded through public health systems, yet access to free vaccination and specific program details vary by country. These variations reflect the diverse healthcare systems and procurement strategies in place, which can impact the systemic approach to HPV prevention. Further harmonization of vaccine procurement and implementation strategies may enhance the effectiveness and equity of HPV vaccination across Europe.

## 1. Introduction

Cervical cancer remains a significant public health challenge, ranking as the fourth most common cancer among women worldwide in 2022 [1]. The primary cause of cervical cancer is persistent infection with high-risk types of human papillomavirus (HPV), making HPV infection a necessary condition for its development [2]. Although HPV infection alone does not always progress to cancer, vaccination against HPV is a key preventative measure that significantly reduces the risk of developing cervical cancer. Advances in vaccination strategies provide a tangible opportunity to achieve large-scale prevention, especially when implemented as part of national immunization programs.

In May 2020, the World Health Organization (WHO) introduced the “Global Strategy to Accelerate the Elimination of Cervical Cancer as a Public Health Problem”. This ambitious initiative aims to eliminate cervical cancer as a population-level issue by the end of this century. The strategy outlines a pathway for reducing the global incidence of cervical cancer to fewer than four cases per 100,000 women annually. To achieve this, one of the key targets is ensuring that by 2030, 90% of girls are vaccinated against HPV before they reach the age of 15 [3]. As of 2024, the WHO reports that HPV vaccination has been fully incorporated into the National Immunization Programs (NIPs) of 137 member countries [4].

In Europe, both the European Cancer Organization (ECO) and the European Union (EU) have reinforced the urgency of implementing comprehensive HPV vaccination programs. The European Beating Cancer Plan calls for all EU member states to adopt gender-neutral vaccination strategies and monitor the progress of these programs. The ECO has set an ambitious goal that, by 2030, all European countries should have HPV vaccination programs in place, with a target of achieving at least 90% vaccination coverage among adolescents of both sexes [5]. The European Center for Disease Prevention and Control (ECDC) also provides an important platform for monitoring recommended vaccination schedules across selected European countries [6]. However, existing summaries from the ECDC and other resources do not always capture the full details of how free HPV vaccination programs are being implemented in all EU member states.

Currently, two types of HPV vaccines are available: a two-valent recombinant vaccine with the Cervarix adjuvant targeting HPV types 16 and 18, and a nine-valent recombinant Gardasil 9 vaccine, which targets HPV types 6, 11, 16, 18, 31, 33, 45, 52, and 58 [2] and has replaced the earlier four-valent Gardasil/Silgard formulation [7,8].

By 2022, various organizations and scientific societies (e.g., the Centers for Disease Control and Prevention—CDC 2021 [9], National Cancer Institute—NCI 2021 [10]) recommended following the SmPC schedule for HPV vaccination: a two-dose schedule for children under 15 years of age and a three-dose schedule for individuals 15 years and older, as well as for children with immunodeficiencies. In 2022, the WHO [11] became the first organization to suggest that a single dose of the HPV vaccine could be used, which is beneficial from a public health standpoint, offering comparable individual protection to the two-dose regimen and being simpler to implement.

To better understand the variations in HPV vaccination programs across the EU, this study aims to review the latest available information on nationwide population-based HPV vaccination initiatives and the selection criteria for vaccine procurement in different countries. This analysis provides insights into the diverse approaches to HPV prevention within the EU, which reflect the broader challenges and opportunities in achieving the elimination of cervical cancer across Europe.

The aim of this study is to analyze and compare the principles of implementing national HPV vaccination programs across European Union countries, with a particular focus on access to free vaccination and the selection criteria for vaccine procurement.

## 2. Method

This analysis of HPV vaccination program implementation across EU countries was conducted through a detailed review of publicly available information from official online sources. These sources included the websites of government agencies, public health authorities, and specialized portals dedicated to vaccination-related information specific to each member state. The selection of these sites ensured access to the most reliable, accurate, and up-to-date data on national HPV vaccination policies and practices. By prioritizing official and authoritative platforms, we aimed to provide a robust foundation for understanding the diverse approaches to HPV vaccination across the EU. To conduct the search, we utilized the “Google” search engine (Google LLC, Mountain View, CA, USA) [12], employing a systematic approach with terms related to HPV vaccination (such as “HPV vaccination”, “HPV vaccination program”, and “HPV National Program”) in the official languages of all EU member states. This multilingual strategy enabled us to capture country-specific nuances in vaccination policies and practices. Whenever English-language content was available, it was prioritized for review to maintain clarity and reduce translation-related discrepancies. However, in instances where English versions of the sources were unavailable, we used “Google Translate” (Google LLC) [13] to convert the content into English. This ensured that the data from non-English sources were comprehensible and consistent with the rest of the analysis. By employing this comprehensive and inclusive methodology, we effectively mitigated language barriers, allowing for a thorough comparison of HPV vaccination strategies across EU countries. This approach underscores the commitment to accuracy and inclusivity in data collection, facilitating meaningful insights into the implementation of HPV vaccination programs.

The websites were searched by one author (W.M.) and verified by the next (T.T. and U.R.). Data analyses regarding the rules for implementing vaccination programs in each subsequent country began with a search for information on official government websites in domains intended for government organizations (.gov), which guaranteed their reliability. In the next step, official national organizations/agencies dealing with public health in a given country were searched. Subsequently, dedicated websites regarding vaccination programs were used, after verifying whether these were official websites to which the aforementioned government websites redirect.

While reviewing the websites, the following information was sought regarding the current rules for receiving free HPV vaccinations in a given country:

- Gender of the eligible population;
- Age of the eligible population;
- Financed vaccine preparation;
- Vaccination administration regimen used;
- Possible comments (e.g., additional conditions related to financing vaccinations or information on when the current vaccination rules were introduced).

Data were collected in an Excel directory according to the categories established above.

Additionally, in order to analyze the selection criteria for vaccine procurement for suppliers of the vaccine products used in national HPV vaccination programs in European Union countries, a search was conducted in Tenders Electronic Daily (TED) (Publications Office of the European Union, Luxembourg). This database is an online version of the “Supplement to the Official Journal of the European Union” on public procurement in Europe, which is a platform providing free electronic access to calls for tenders published by EU institutions, agencies, and other bodies [14]. In order to analyze the most current data, it was decided to conduct a search within the last 3 years, i.e., from 1 January 2021.

Below are the search options used:

- Search scope: All ads.
- Full text: hpv.
- Publication date: from 1 January 2021.
- Search filters used:
  ○ Technology and equipment;
  ○ Medical devices, pharmaceuticals, and personal care products;
  ○ Pharmaceutical products;
  ○ General anti-infectives for systemic use, vaccines, anti-cancer and immunomodulatory agents;
  ○ General anti-infectives for systemic use and vaccines;
  ○ Vaccines;
  ○ European Union.

## 3. Results

Information was found on the rules of implementation of free HPV vaccination in all 27 countries of the European Union. The rules of access to free vaccination for citizens of EU countries depend on individual health care systems, which also affects the systemic approach to the primary prevention of HPV. Some countries have introduced nationwide systems of vaccination for children of a certain age, such as Poland. Another way of implementing HPV prophylaxis is through school vaccination programs covering specific age groups of schoolchildren, e.g., in Hungary, vaccination is free only in the school vaccination campaign. The age of administration of the vaccine is not always identical throughout the country, e.g., in Belgium, the Flemish Community conducts vaccination of students in the first year of secondary school, while in the area of Wallonia and Brussels, HPV vaccination is conducted in the second year of secondary school. Some countries do not have nationwide vaccination campaigns; however, the cost of HPV vaccination is covered by social insurance, e.g., Germany. A summary of the information found is provided in Table 1 below.

### 3.1. Gender of the Target Population

Twenty-six of the twenty-seven European Union countries offer free HPV vaccination for girls and boys. Only the Bulgarian National Program for the Primary Prevention of Cervical Cancer for 2021–2024 provides free vaccinations only to girls.

### 3.2. Age of the Target Population

According to the Summary of Product Characteristics of the vaccines currently authorized and available in the EU (Cervarix [7], Gardasil 9 [8]), these products can be used from the age of nine. Five EU countries have decided to provide financing for vaccinations starting from the lowest age provided by the manufacturer, i.e., Austria, Croatia, Greece, Luxembourg, and Germany.

Ten out of twenty-seven EU countries start funding vaccinations for children from the age of 11.

The highest age of the population covered by free vaccination is currently in Austria, i.e., up to 30 years of age. However, in Romania, HPV vaccinations are covered by a 50% refund in the population of women aged 19 to 45 years.

### 3.3. Vaccine Preparation Available

In the vast majority of EU countries (22/27) it is possible to obtain free vaccination only with the nine-valent HPV vaccine. In the Netherlands and Finland, only the bivalent vaccine is a financed vaccine. In Germany, Poland, and the Czech Republic, it is possible to be vaccinated with both Cervarix and Gardasil 9 free of charge.

### 3.4. Administration Schedule

The manufacturers of both vaccines registered and available in the EU recommend two-dose vaccination schedules for the population up to and including 14 years of age and extending the administration schedule to three doses for people over 15 years of age [41,42]. Most EU countries vaccinate according to registered vaccination schedules. The exceptions are Estonia and Ireland, where national programs assume vaccinating children with only one dose of Gardasil 9, which is not intended but is in line with the latest WHO recommendations [11]. According to the information found, Spain is preparing to introduce a one-dose vaccination regimen from next year, i.e., 2025.

### 3.5. Selection Criteria for Vaccine Procurement

A search in the Tenders Electronic Daily database yielded 75 results, of which 17 advertisements from 10 EU countries concerned the supply of HPV vaccines for nationwide use. The characteristics of the identified advertisements are presented in Supplementary Materials File S1.

The most common selection criterion for selecting a supplier of a vaccine preparation for nationwide vaccination against HPV was the lowest price. Other frequently appearing criteria were the delivery date of the product and the expiry date of the delivered product. Moreover, in the announcement from Poland no. 368495-2023, a quality criterion appeared in the form of “Content of more than 4 serotypes in the vaccine composition” [46], and in the Estonian announcement from 2022, for selecting a bidder, in addition to the price, a criterion in the form of “quality assessment and vaccine effectiveness” was specified [47].

## 4. Discussion

Based on the information found in the review of websites in the domains of European Union countries, the rules for the implementation of free HPV vaccination in all 27 European Union countries were summarized.

On the European Centre for Disease Prevention and Control (ECDC) platform, information is collected on the recommended vaccination schedules in selected European countries; however, the list does not indicate the current rules for conducting free vaccination programs in all the countries presented [6]. Discrepancies in the presented recommended HPV vaccination schedules affect the continuous changes in the approach to the implementation of HPV vaccination across the European Union. For the purpose of this discussion, we searched for current clinical practice guidelines for the implementation of HPV vaccination programs, which also translate into observed trends in the implementation rules of national HPV programs.

The European Cancer Plan stipulates that by 2030, all European countries should have HPV vaccination programs targeting both sexes [5]. Currently, only Bulgaria has not aligned its vaccination program with the recommendation to extend vaccination to boys as well.

In the most recent 2023 recommendations, the Australian Government Department of Health [48], the Joint Committee on Vaccination and Immunization [49], and the Royal College of Physicians of Ireland/National Immunization Advisory Committee [50] explicitly recommend the use of Gardasil 9 as part of population vaccination. The trend of more frequent recommendations of vaccination with the nine-valent formulation can also be seen in the compiled summary of vaccination programs in EU countries, where Gardasil 9 is used in the vast majority of national programs.

When it is clear which vaccine formulation is used in a national program, the main criterion for tenders for the supply of vaccines is price, which, according to an analysis by Qendri in 2019 [51], may continue to have a positive impact on the decrease in average tender prices, thus reducing the cost of running population-based vaccination programs.

Another interesting phenomenon that can be seen in the national programs of EU countries is the transition of the first EU countries to an off-label single-dose regimen for HPV vaccination. Countries that have introduced this regimen have cited the current WHO recommendation of 2022, in which the World Health Organization indicates that, based on high-quality RCTs, a single dose of p/HPV vaccine is found to have very high efficacy (97.5%) in girls under 20 years of age. This makes the recommendation of a single-dose vaccination regimen seem reasonable from a public health perspective, as it provides a comparable and individual level of protection while being easier to implement than a two-dose schedule. In addition, the implementation of a single vaccination schedule may allow the vaccine to reach more girls more quickly, thus leading to the faster development of community immunity [11].

Incoming new data on the efficacy of HPV vaccines are influencing changes or refinements in the clinical recommendations of scientific societies. Changes in recommendations, combined with differences in health care systems across EU countries, mean that the approach to the principles of implementing free HPV vaccination continues to evolve, and a uniform standard for nationwide HPV disease prevention has not been developed.

## 5. Conclusions

In all EU countries, HPV vaccination is funded through public health systems, but access to free vaccination varies according to the structure of each nation’s healthcare system. These differences influence the overall approach to HPV primary prevention, particularly in terms of accessibility and implementation strategies. Notably, most EU countries have adopted gender-neutral vaccination policies, ensuring that both girls and boys are eligible for free HPV vaccination. This harmonized approach extends to the selection of vaccine products, with the majority of countries using the nine-valent HPV vaccine, leading to consistent selection criteria for vaccine procurement across the EU.

However, a significant challenge remains in achieving high vaccination uptake, which is crucial for the goal of eradicating cervical cancer. To meet this objective, it is essential to maximize vaccination coverage across all eligible populations. Further research into the effectiveness of simplified vaccination schedules, such as single-dose regimens, may support this effort by making vaccination programs more accessible and efficient. Additionally, intensified public health campaigns focusing on education and awareness about the risks of HPV infection and the benefits of vaccination will be critical in increasing vaccine acceptance and uptake. By addressing these challenges, EU countries can move closer to the shared goal of eliminating cervical cancer.

## Figures and Tables

**Table 1:** Overview of the rules for implementing free HPV vaccinations in European Union countries.

Country	Girls/Boys	Age	Vaccine	Number of Doses in Vaccination Schedule	Required Interval Between Doses	Additional Notes
Austria [15,16]	Girls and boys	9–21 * years	Gardasil 9	2 doses	6–12 months	Conditions as of 1 February 2023 From age 15 to 21, the 2-dose schedule is used off-label and recommended according to the 2023 Austrian vaccination plan. For individuals aged 21 and older, a 3-dose HPV vaccination schedule is recommended. * Free vaccination may be extended up to age 30 from April
Belgium [17]	Girls and boys	11–13 years *	Gardasil 9	2 doses	5–13 months	As of 1 January 2024 1st year of secondary school (12 years)—Flemish Community; 2nd year of secondary school (13 years)—Wallonia-Brussels Federation
Bulgaria [18,19]	Girls	10–13 years	Gardasil 9	2 doses	6 months	-
Croatia [20]	Girls and boys	9–25 years *	Gardasil 9	Up to 14 years old: 2 doses; 15+ years: 3 doses	6 months/0–2–6 months	Vaccination at school in grades 5–8 of primary school
Cyprus [21]	Girls and boys	11–13 years	Gardasil 9	2 doses	6 months	Conditions as of 1 January 2024
Czechia [22,23]	Girls and boys	11–15 years	Cervarix or Gardasil 9	2 doses	6–12 months	Conditions as of January 2024, * public health insurance reimbursement
Denmark [24]	Girls and boys	12–18 years	Gardasil 9	Up to 14 years: 2 doses; 15+ years: 3 doses	5–13 months/0; 1–2; 3–4 months	-
Estonia [25,26]	Girls and boys	12–18 years	Gardasil 9	1 dose (3 doses for immunocompromised individuals)	-	Conditions as of February 2024
Finland [27]	Girls and boys	10–12 years	Cervarix	Up to 15 years: 2 doses; 15+ years: 3 doses	5 months/0–1–6 months	-
France [28]	Girls and boys	11–14 years *	Gardasil 9	2 doses	6 months	Conditions include the 2023/2024 school year * Vaccination campaign for 5th-grade students
Greece [29]	Girls and boys	9–11 years	Gardasil 9	Up to 15 years: 2 doses; 15+ years: 3 doses	6 months/0; 1–2; 6 months	-
Spain [30]	Girls and boys	12 years *	Gardasil 9	2 doses **	6 months	* Catch-up until 18 years old
						** Starting in 2025: 1 dose
Ireland [31]	Girls and boys	12 years *	Gardasil 9	1 dose (3 doses for immunocompromised individuals)	-	Vaccination for boys introduced in September 2019; * in the first year of secondary school
Lithuania [32]	Girls and boys	11 years	Gardasil 9	2 doses	6 months	Conditions as of February 2023
Luxembourg [33]	Girls and boys	9–14 years; catch-up until 20 years old	Gardasil 9	2 doses	6 months	Vaccination for individuals aged 21 and older is available as a catch-up vaccination but is not part of the free national program. Three-dose schedule: 0, 2, 6 months
Latvia [34]	Girls and boys	12–17 years	Gardasil 9	2 doses (3 doses for immunocompromised individuals)	6 months	Conditions as of 2 May 2023
Malta [35]	Girls and boys	12–13 years	Gardasil 9	2 doses	6–12 months	-
Netherlands [36]	Girls and boys	10–18 years	Cervarix	2 doses *	6 months	Conditions as of 2022; only individuals with weakened immune systems, such as those with autoimmune diseases or HIV, are eligible for 3 doses
Germany [37]	Girls and boys	9–14 years; catch-up until 18 years old	Cervarix or Gardasil 9	Up to 14 years: 2 doses; 15+ years: 3 doses	at least 5 months	No national program—costs covered by social insurance
Poland [38]	Girls and boys	11–14 years	Cervarix or Gardasil 9	2 doses	6–12 months	Conditions as of 1 June 2023
Portugal [39]	Girls and boys	10 years	Gardasil 9	2 doses	6 months	Conditions as of 1 October 2020
Romania [40]	Girls and boys	11–19 years	Gardasil 9	2 doses	6 months	Conditions as of September 12, 2023; 50% reimbursement for women aged ≥19 and ≤45 years
Slovakia [41]	Girls and boys	12–15 years	Gardasil 9	2 doses	6 months	Conditions as of 1 December 2023
Slovenia [42]	Girls and boys	11 years *	Gardasil 9	2 doses	6 months	* Vaccination in 6th grade of primary school; in the 2023/24 school year, also for boys in 1st and 3rd years of secondary school
Sweden [43]	Girls and boys	11 years *	Gardasil 9	2 doses (3 doses for 15+ years or immunocompromised individuals)	6 months/0; 1–2; 4–5 months	* Vaccination in 5th grade
Hungary [44]	Girls and boys	12 years *	Gardasil 9	2 doses	6 months	* Vaccination in 7th grade of primary school
Italy [45]	Girls and boys	11+ years	Gardasil 9	2 doses (3 doses for individuals aged 15+)	6 months	-

## Data Availability

All data are available from the corresponding author.

## Supplementary Materials

The following supporting information can be downloaded at: https://www.mdpi.com/article/10.3390/vaccines12121325/s1, File S1: Information contained in tender notices found for the provision of p/HPV vaccines.
