# Supplementary material for: Analysis of Implementation Strategies for Nationwide HPV Vaccination Programs Across European Union Countries
Source: Vaccines (Basel). 2024 Nov 26;12(12):1325. doi: 10.3390/vaccines12121325 (PMC11680320; doi:10.3390/vaccines12121325)
Supplement: Supplementary file 1 [file vaccines-12-01325-s001.zip › vaccines-3299900-supplementary.pdf]

File S1. Information contained in the found tender notices for the supply of anti-HPV vaccines.

Country/territory	Advertisement No (announcement date)	Title of the tender	Tender description	Criteria of choice	Number of offers	Selected bidder
**France(Department of Nièvre)**	73658-2024 (05/02/2024)	Obtaining and delivering HPV vaccines	As part of the vaccination campaign against papillomavirus, the Faculty Council is launching consultations on the purchase and delivery of these vaccines to students and health centers.	• Other (specified in consultation documents)	n.d	n.d
**Poland**	647329-2023 (25/10/2023)	Human papillomavirus (HPV) vaccine (recombinant, adsorbed in a single dose of 0.5 ml) - 9-valent	The subject of the order is the supply of a vaccine against human papillomavirus (HPV) (recombinant, adsorbed in a single dose of 0.5 ml) - 9-valent for use in the universal HPV vaccination program	• Quality criterion - Vaccine validity period (Weight: 40)<br>• Price – (Weight: 60)	n.d	n.d
**France (Alpes Maritime Department)**	677728-2023 (08/11/2023)	Obtaining a 9-valent vaccine against human papillomavirus (HPV) for public vaccination centers as part of a nationwide campaign at universities	Obtaining a 9-valent vaccine against human papillomavirus (HPV) for public vaccination centers as part of a nationwide campaign at universities.	• Quality criterion – technical value (Weight: 30)<br>• Price (Weight: 70)	1	Laboratoire MSD France
**Finland**	593235-2023 (02/10/2023)	Human Papillomavirus Vaccine (HPV) 2024-2026 (2026-2027 and 2027-2028)	The Ministry of Social Affairs and Health and the Finnish Institute of Health and Welfare request to submit a tender for a vaccine against human papillomavirus included in the National Vaccination Program in Finland.	• Price<br>• Quality	n.d	n.d
**Poland**	567678-2023 (20/09/2023)	Human papillomavirus (HPV) vaccine (recombinant, adsorbed in a single dose of 0.5 ml) - 9-valent	The subject of the order is the supply of a vaccine against human papillomavirus (HPV) (recombinant, adsorbed in a single dose of 0.5 ml) - 9-valent for use in the universal HPV vaccination program.	• Quality criterion - Vaccine validity period (Weight: 40)<br>• Price – (Weight: 60)	n.d	n.d
**Hungary**	494584-2023 (14/08/2023)	Purchasing the HPV vaccine	For the years 2023-2025, in accordance with the government's decision, the tenderer purchases a 9-component, 2-vaccine vaccine against cervical cancer, a	• Price	1	MSD Pharma Hungary Limited

Country/territory	Advertisement No (announcement date)	Title of the tender	Tender description	Criteria of choice	Number of offers	Selected bidder
			recombinant, adsorbed vaccine against human papillomavirus for vaccinations due at the age of 12.			Liability Company
**Sweden**	456478-2023 (28/07/2023)	HPV vaccine in accordance with the national childhood vaccination program 2021	The procurement covers human papillomavirus (HPV) vaccines, which are mainly used as part of the general childhood vaccination program and meet the needs of the contracting authorities as described in this procurement document.	• Price	1	Merck Sharp & Dohme (Sweden) AB
**Austria**	413560-2023 (10/07/2023)	HPV vaccine	The subject of this framework agreement is the supply of HPV vaccines with a medicinal product authorization valid in Austria in accordance with the Austrian Medicines Act or the EU basic legal standards regarding a jointly implemented child vaccination program at federal, state and social level for authorized bodies as well as for the Republic of Austria (Government federal) and all other customers according to the customer list attached to the tender documents, all represented in the procurement process by Bundesbeschaffen GmbH.	• Price	1	Merck Sharp & Dohme Gesellschaft mbH
**Poland**	368495-2023 (21/06/2023)	Recombinant, adsorbed vaccine against human papillomavirus (HPV) for girls and boys	The subject of the order is the supply of a recombinant, adsorbed vaccine against human papillomavirus (HPV) for girls and boys; single dose administered to the patient in the vaccination schedule - 0.5 ml.	• Part 1 of the order:<br>• Quality criterion - Vaccine validity period (Weight: 40)<br>• Price – (Weight: 60)<br><br>• Part 2 of the order:<br>• Quality criterion - The vaccine contains more than 4 serotypes / Weight: 70	Lot 1 of the order: 1<br><br>Lot 2 of the order: 1	Part 1 of the order: GSK Services Sp. z o. o<br><br>Part 2 of the order:<br>Urtica Sp. z o. o

Country/territory	Advertisement No (announcement date)	Title of the tender	Tender description	Criteria of choice	Number of offers	Selected bidder
				• Price - Weight: 30		
**Finland**	673320-2022 (02/12/2022)	Human Papillomavirus Vaccine (HPV) 2020-2021 (2022-2023)	The Ministry of Social Affairs and Health and the Finnish Institute of Health and Welfare invite you to submit an offer for a vaccine against human papillomavirus included in the National Vaccination Program in Finland.<br>The contract period is approximately two years 2020-2021. The order provides for two options for extending the contract for 2022 and 2023.	• Price	1	GlaxoSmithKline Oy
**Denmark**	373023-2022 (11/07/2022)	Tender for a framework agreement for the supply of HPV vaccine 2022-2025	A framework contract is offered for the purchase of HPV vaccine for the Danish childhood vaccination program. The vaccine must contain at least human papillomavirus types 16, 18, 31, 33, 45, 52 and 58. The offered vaccine must have a central (EMA) or national (Medical Medicines Agency) vaccine approval valid in Denmark at the time of submission of the offer.	• Price	1	MSD Danmark ApS
**Belgium(Flanders)**	304738-2022 (07/06/2022)	Government contract for the supply of HPV vaccines for the vaccination program in Flanders	Government contract for the supply of HPV vaccines for the vaccination program in Flanders	• Price	1	MSD Belgium
**Estonia**	220701-2022 (27/04/2022)	Purchasing a vaccine against human papillomavirus	A public procurement has been prepared for the purchase of a vaccine against human papillomavirus (HPV). Tenders submitted must comply with the technical specifications set out in the Annex	• Assessment of vaccine quality and effectiveness (Weight: 70.0)<br>• Price – (Weight: 30.0)	1	TAMRO EESTI OÜ
**Romania**	207254-2022 (19/04/2022)	HPV 2 vaccine delivery	HPV vaccine delivery	• Product delivery date (Weight: 10)<br>• Product expiry date (Weight: 10)	4	MEDIPLUS EXIM

Country/territory	Advertisement No (announcement date)	Title of the tender	Tender description	Criteria of choice	Number of offers	Selected bidder
				• Price – (Weight: 80)		
**Hungary**	523076-2021 (15/10/2021)	Purchasing the HPV vaccine	Under the sales agreement, the Offeror purchases a 9-component, 2-vaccine vaccine against HPV for the 7th Primary School. for adolescent boys who finish grade (7th grade) and are 12 years old, and for girls.<br>The bidder provides for the possibility of submitting partial offers according to the following parts:<br>Part 1: HPV vaccine for adolescent boys, quantity: 55,000 doses + 55,000 dose option<br>Part 2: HPV vaccine for adolescent girls, quantity: 100,000 doses + 40,000 dose option	• Price	Lot 1 of the order: 1<br>Lot 2 of the order: 1	Part 1 of the order: MSD Pharma Węgry Spółka z ograniczoną odpowiedzialnością<br>Part 2 of the order:<br>MSD Pharma Hungary Limited Liability Company
**Romania**	235500-2021 (11/05/2021)	HPV vaccine delivery	HPV vaccine delivery	• Product delivery date (Weight: 10)<br>• Product expiry date (Weight: 10)<br>• Price – (Weight: 80)	5	MEDIPLUS EXIM
**Hungary**	72575-2021 (12/02/2021)	Purchase of the HPV vaccine for adolescent boys	As part of the sales contract, the Bidder purchases a vaccine against the HPV virus, Primary School VII. for adolescent boys who have completed grade (7th grade) and are 12 years old.	• Price	1	MSD Pharma Hungary Limited Liability Company
